# The Paradoxical Behavior of microRNA-211 in Melanomas and Other Human Cancers

**DOI:** 10.3389/fonc.2020.628367

**Published:** 2021-02-08

**Authors:** Animesh Ray, Haritha Kunhiraman, Ranjan J. Perera

**Affiliations:** ^1^ Riggs School of Applied Life Sciences, Keck Graduate Institute, Claremont, CA, United States; ^2^ Division of Biology and Biological Engineering, California Institute of Technology, Pasadena, CA, United States; ^3^ Cancer & Blood Disorder Institute, Johns Hopkins All Children’s Hospital, South, St. Petersburg, FL, United States; ^4^ Department of Oncology, Sidney Kimmel Comprehensive Cancer Center, School of Medicine, Johns Hopkins University, Baltimore, MD, United States

**Keywords:** microRNA-211, melanoma, tumor-promoter, tumor suppresser, epigenetics, miRNA, bistability, MIR211

## Abstract

Cancer initiation, progression, and metastasis leverage many regulatory agents, such as signaling molecules, transcription factors, and regulatory RNA molecules. Among these, regulatory non-coding RNAs have emerged as molecules that control multiple cancer types and their pathologic properties. The human microRNA-211 (MIR211) is one such molecule, which affects several cancer types, including melanoma, glioblastoma, lung adenocarcinomas, breast, ovarian, prostate, and colorectal carcinoma. Previous studies suggested that in certain tumors MIR211 acts as a tumor suppressor while in others it behaves as an oncogenic regulator. Here we summarize the known molecular genetic mechanisms that regulate *MIR211* gene expression and molecular pathways that are in turn controlled by MIR211 itself. We discuss how cellular and epigenetic contexts modulate the biological effects of MIR211, which exhibit pleiotropic effects. For example, up-regulation of *MIR211* expression down-regulates Warburg effect in melanoma tumor cells associated with an inhibition of the growth of human melanoma cells *in vitro*, and yet these conditions robustly increase tumor growth in xenografted mice. Signaling through the DUSP6-ERK5 pathway is modulated by MIR211 in BRAF^V600E^ driven melanoma tumors, and this function is involved in the resistance of tumor cells to the BRAF inhibitor, Vemurafenib. We discuss several alternate but testable models, involving stochastic cell-to-cell expression heterogeneity due to multiple equilibria involving feedback circuits, intracellular communication, and genetic variation at miRNA target sties, to reconcile the paradoxical effects of MIR211 on tumorigenesis. Understanding the precise role of this miRNA is crucial to understanding the genetic basis of melanoma as well as the other cancer types where this regulatory molecule has important influences. We hope this review will inspire novel directions in this field.

## Introduction

MicroRNAs^1^ (miRNAs) are highly conserved small non-coding RNA molecules of approximately 22 nucleotides that control gene expression either by direct translational inhibition of protein synthesis or by affecting the degradation of the target mRNA (2). miRNA biogenesis occurs through biochemical pathways that are well conserved among metazoans (3), which is described briefly below [miRNA biogenesis has been reviewed recently (4)]. In animals, miRNA transcription begins by the binding of RNA polymerase II enzyme (Pol II) to transcriptional regulatory regions. The primary pol II transcript (Pri-miRNA) molecules, which are precursors of the mature miRNAs, often contain multiple and complex intramolecularly bases-paired looped structures and are subsequently processed by the nuclear enzyme Drosha to produce the intermediate precursor molecules termed Pre-miRNA. Pre-miRNA molecules generally possess a single hairpin loop. Pre-miRNAs are then exported to the cytoplasm by Exportin-5 (Ex-5) and are further processed to generate a mature miRNA duplex without a hairpin by the Dicer enzyme. Mature miRNA molecules, which can be either the 5′ (miRNA-5p) or the 3’ (miRNA-3p) component of the Pre-miRNA double-stranded stems of the hairpin, are loaded into the multi-protein RNA-induced silencing complex (RISC) to generate single-stranded guide miRNAs. Such guide miRNA molecules preloaded into the RISC complex interact with various target mRNAs by complementary base pairing over short regions of perfect or near-perfect sequence complementarity, most frequently at the 3’untranslated regions (3’UTR) of the target mRNAs (4). In animals, long 3’UTRs of target mRNAs are often recognized by multiple miRNAs (5, 6). Most such mRNAs with long 3’UTR are known to be involved in cellular fate-determination and developmental processes (4, 7), whereas target genes with relatively short 3’UTR regions generally encode proteins that participate in fundamental cellular biochemistry and stress response (7, 8). For example, genes encoding ribosomal RNAs and proteins have shorter 3’UTR sequences; therefore, these RNA molecules present less complex regulatory potential through the binding of a small number of different miRNAs than do genes encoding longer 3’UTRs, which are generally associated with mRNA of regulatory proteins. Less frequently, biologically important miRNA binding sites are also located in 5’UTR regions, coding regions and promoter regions of coding genes (9, 10). This dichotomy in 3’UTR or 5’UTR lengths have dynamical consequences: genes encoding proteins that participate in fundamental biochemistry/metabolism of the cells (“housekeeping” genes) are regulated quantitatively, as a rheostat does, through a graded response to regulation by miRNAs (11). By contrast, genes encoding regulatory proteins such as transcription factors, each exhibiting multiple miRNA targets, are regulated through complex analog-logical circuits that often result in a switch-like behavior in their expression levels. To understand the biological effects of a miRNA, therefore, it is important to understand how the expression of the miRNA is regulated as well as how that specific miRNA molecule regulates other genes. We illustrate some of these complexities with MIR211, which is one of the major regulators in human cancer, specifically in human melanomas (12–17). Specifically, this molecule can act both as a tumor suppressor and an oncogene based on the cellular context and thus demonstrates a paradoxical behavior in melanoma and other cancers.

## Direct Transcriptional Regulation of *MIR211* Expression

*MIR211* gene is located on chromosome 15q13.3, encoded within the sixth intron of the *Transient Receptor Potential cation channel subfamily M member1* gene (*TRPM1*, Melastatin). The primary human melanocytes exhibit a high expression level of MIR211 (12). Normal human organs such as the eye (18) and the heart (19) also exhibit high expression levels of the primary *TRPM1* transcript. As expected, the levels of *TRPM1* and *MIR211* expression are positively correlated (20).

**Figure 1:**
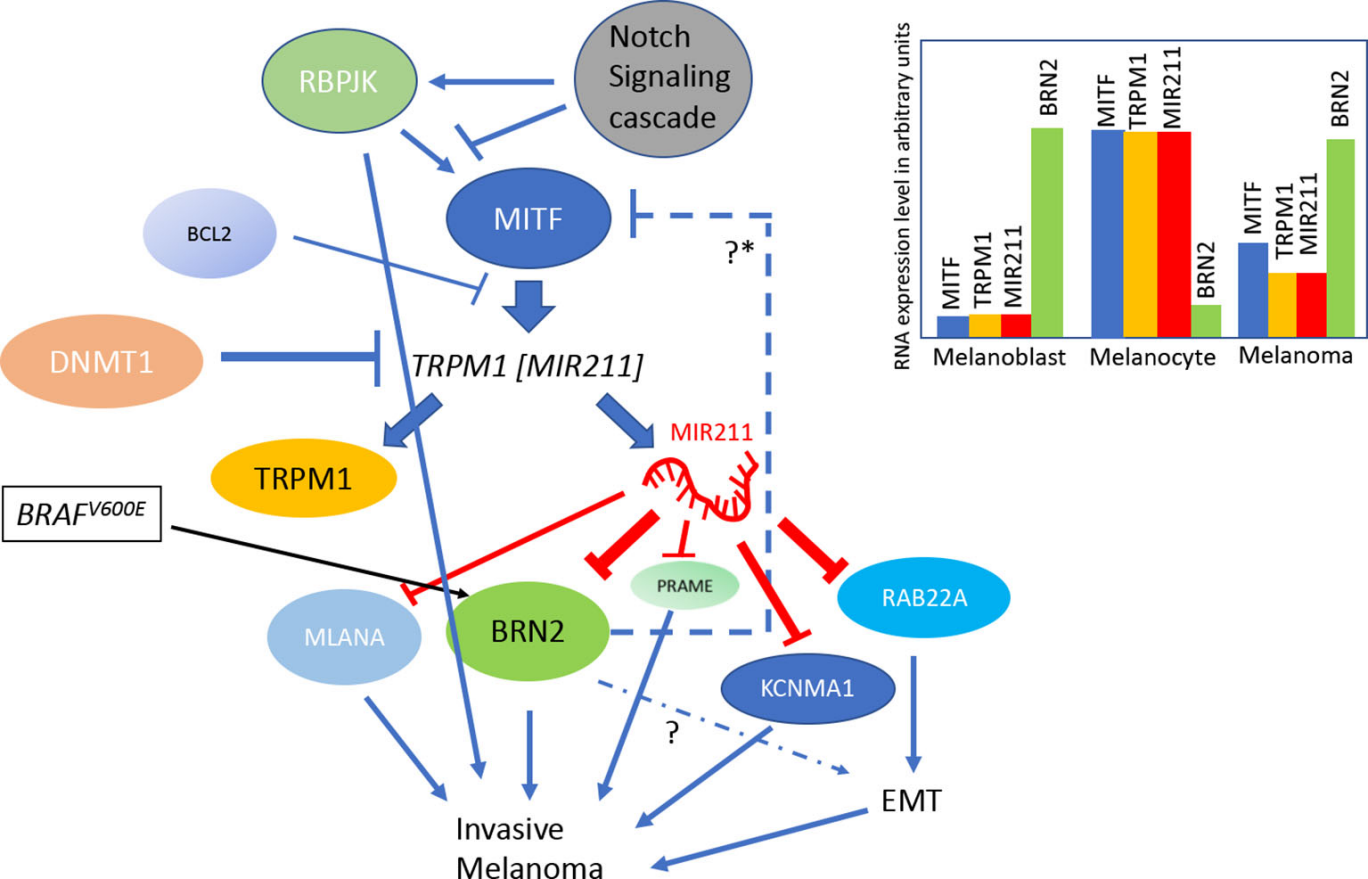
A model summarizing the regulation of MIR211 gene expression: *MIR211* gene is nested within the *TRMP1* transcript. *TRPM1* transcription is promoted by the transcription factor MITF, and the latter’s transcription is in turn regulated by the Notch signaling pathway *via* the expression of RBPJK. The DNA methyl transferase DNMT1 regulates the epigenetic state of *TRPM1* gene *via* promoter methylation. MIR211 negatively regulates various genes, including MLANA, PRAME, RABB22A, KCNMA1, and BRN2. Simultaneously *BRN2* gene product induces *MIR211*expression through an unknown mechanism. Apart from that, *MIR211* expression also negatively regulated by pro-apoptotic protein BCL2 through MITF inhibition. The BRAFV600E mutation, a frequent driver mutation for melanoma, activates BRN2, which is negatively regulated by MIR211. EMT is Epithelial Mesenchymal Transition. The inset is a color-coded summary of salient mRNA expression patterns in different cell types related to melanoma formation (blue, MITF; orange, TRPM1; red, MIR211; green, BRN2). The Y-axis is an arbitrary scale of gene expression levels, used as a schematic representation of the salient points on how *MIR211* expression varies in various cell types. See text for evidence regarding these circuits and the gene expression levels from the following sources (12, 14, 25–33). *Proposed negative regulation.

*MIR211* has now been recognized as an important player in the molecular pathogenesis of skin cancers. The Melanocyte Inducing Transcription Factor (MITF) regulates *MIR211*’s host gene *TRPM1* by serving as its primary transcriptional regulator. MITF is one of the critical regulators of melanocyte differentiation and melanoma formation (12, 21, 22). A positive correlation of gene expression levels between *MIR211* and other MITF target genes such as *TYRP1, TYR, MLANA, CDK2*, and *SILV* has also been reported, further supporting the role of MITF in regulating *MIR211* expression (12, 23), and allowing the inclusion of *MIR211* into the gene ontology (24) cluster of “melanosome pigment granule related genes” (FDR corrected *P* =4.36 × 10^−8^). An *MITF-MIR211-BRN2* regulatory feedback loop has been demonstrated (**Figure 1**), and this regulatory mechanism may be important for cell state specification in both melanoblasts and melanoma cells (25–28). BRN2 (Brain-Specific Homeobox/POU Domain Protein class 3 transcription Factor 2 or POU3F2), a transcription factor, is associated with aggressive melanoma development, and MIR211 is a strong suppressor of *BRN2* mediated invasiveness (25). In this feedback model BRN2 is a direct target of MIR211: predictably, BRN2 expression shows an inverse correlation with *MITF* expression. On the other hand, BRN2 has been shown to be a repressor of *MITF* transcription (29). This is predicted to induce bistable states in different subpopulations of cells (21): one in which *MIR211* expression and in the other BRN2 expression would dominate, thus producing different cellular behaviors due to mutually exclusive expression of these two genes in the two subpopulations (see later for a significance of this feedback loop).

The transcription regulatory gene *Recombination signal Binding Protein for immunoglobulin kappa J region* (*RBPJ*), one of the primary downstream molecular effectors of Notch signaling, shares a similar gene expression profile with that of MITF, suggesting a conserved biological function (30). Indeed, the expression levels of *RBPJ* and *MITF* exhibit a positive correlation (R=0.47, *P<*10^−10^) in the Genotype-Tissue Expression (GTEx) datasets for healthy skin (both sun-exposed and unexposed), but this correlation is absent in melanoma cells (34). Furthermore, *RBPJ* occupies and regulates the *TRPM1* promoter in a *MITF* dependent manner and thereby increases the expression of *MIR211* (30, 31). Bcl-2, a pro-apoptotic protein, regulates the expression of *MIR211* by modulating *MITF* expression in melanoma cell lines, in addition to the reciprocal regulation of *BCL2* by MITF (32). **Figure 1** summarizes these pathways of regulation of *MIR211* gene.

## The Epigenetic Regulation of *MIR211*

Transcriptional silencing associated with methylation of CpG islands is an important correlate that is thought to be responsible for the regulation of tumor suppressor genes (35–37). DNA methyltransferase 1 (DNMT1) mediated methylation of *TRPM1/MIR211* promoter reduces *MIR211* expression levels in melanoma cells (26). Decreased MIR211 levels were correlated with increased cell proliferation and Epithelial to Mesenchymal Transition (EMT, a morphogenetic state transition that is important for the development of numerous cancer types) of melanoma cells (26). The 3’UTR of the oncogenic *Ras-related protein Rab-22A* (*RAB22A*) gene’s mRNA was found to be a direct target MIR211 (33) suggesting that the suppressive effects of MIR211 on oncogenic properties might be at least partially through the inhibition of RAB22A expression. Similarly, epigenetic silencing of *TRPM1/MIR211* gene by EZH2-induced (Enhancer Of Zeste Homolog 2 protein) histone H3K27 trimethylation and DNMT1 mediated promoter methylation were associated with cell proliferation, increased AKT/β-catenin signaling, and the induction of EMT, whereas restoration of *MIR211* expression reduced cell proliferation, inhibited AKT/β-catenin signaling and reversed EMT in glioblastoma multiformi (38). Thus, the EZH2/DNMT1/MIR211/RAB22A axis might provide novel insights into the molecular pathogenesis of both melanoma and glioblastoma, particularly on the EMT processes in these two different cancers. In a separate study, Li N *et al.* reported that the silencing of *MIR211* expression by methylation is associated with reduced cisplatin sensitivity in melanoma (39). This observation, however, may be interpreted as an indirect consequence of reduced EMT in MIR211 deficient cells, because increased EMT increases the resistance of cells to cisplatin (40).

## Tumor Suppressor Effects of *MIR211*

As expected of a regulatory molecule with a potential tumor-suppressor activity, MIR211 was first identified as one of the most differentially under-expressed miRNA molecules in non-pigmented human melanoma cell lines and in a majority of clinical melanoma tissue samples relative to those expressed in normal melanocytes or in matched normal patient tissues, respectively (12, 14). Conversely, when MIR211 levels were increased artificially in melanoma cells, a significant inhibition of growth and *in vitro* invasive properties were observed, which could again be reversed by inhibiting MIR211 by an antagomir (12–14), fulfilling the criteria for a tumor-suppressor molecule. Consistently, several MIR211 targets genes, all of which were previously known to be oncogenic, were identified, including the *calcium-activated potassium channel subunit a-1* (*KCNMA1*) (12), *Insulin-like Growth Factor 2 Receptor* (*IGF2R*) (14), *TGF-beta Receptor 2* (*TGFBR2*) (41), *Insulin Growth Factor Binding Protein 5* (*IGFBP5*) (42, 43), *POU domain-containing transcription factor BRN2* (25), and *Nuclear Factor of Activated T-cells 5* (*NFAT5*) (14). These evidences together are consistent with the proposal that MIR211 exerts its tumor suppressor properties by means of direct negative regulation of potentially oncogenic mRNAs (44). This proposal is also consistent with the tumor suppressor role of *MITF* as a directly activating transcription factor of *MIR211* gene (12). Subsequently, a series of additional evidences solidified the proposal of miR-211 action as akin to a tumor suppressor for melanomas (13) as well as several other cancer types, including epithelial ovarian cancer (45), ovarian carcinoma (46), breast cancer (47), hepatocellular carcinoma (48), renal cancer (49), and thyroid tumors (50), among others (see **Table 1** for a more complete list).

**Table 1:** Summary of MIR211 target genes and their roles in various cancer types.

	TUMOR/CANCER TYPE	TARGET GENES	ASSOCIATED DYSREGULATED MECHANISMS	REFERENCES
1	MELANOMA	*BRN2 (POU3F2)* *PDK4* *TGFBR2* *NFAT5* *RAB22A* *IL6* *IGF2R* *BCL2* *EDEM* *IGFBP5* *AP1S2* *SOX11* *SERINC3*	INVASION CELL GROWTH INVASION INVASION EMT INVASION CELL GROWTH APOPTOSIS PIGMENTATION	(14, 25, 26, 32, 51–53)
2	MALIGANANT MELANOMA	*PRAME*		(54)
3	MULTIPLE MYELOMA	*CHOP*	APOPTOSIS	(55)
4	THYROID CANCER	*SOX11*	APOPTOSIS	(50)
5	EPITHELIAL OVARIAN CANCER	*CYCLIN D1CDK1*	CELL CYCLE, APOPTOSIS	(45)
7	CERVICAL CANCER	*MUC4* *SPARC* *ZEB1*	INVASION & EMT EMT PROLIFERATION AND METASTSIS	(16, 56, 57)
10	GASTRIC CANCER	*MMP-9* *SOX4*	EMT CANCER METASTASIS	(58, 59)
12	BLADDER CANCER	*SNAI1*	CANCER METASTASIS	(60)
13	OVARIAN CANCER	*PHF-19*	PROLIFERATION, MIGRATION AND APOPTOSIS	(46)
15	GLIOBLASTIOMA	*MCM3AP-AS1* *KLF5*	ANGIOGENESIS ANGIOGENESIS	(61)
17	HEPATOCELLULAR CARCINOMA	*SATB2* *SPARC*	PROLIFERATION AND INVSION	(48, 62)
19	BREAST CANCER	*CDC25B* *CCNB1* *FOXM1*	PROLIFERATION, MIGRATION AND INVASION	(63)
22	COLORECTAL CANCER	*CDH5*	PROLIFERATION AND MIGRATION	(64)
23	ORAL SQUAMOUS CANCER	*BIN1*	PROLIFERATION, MIGRATION AND INVASION	(65)
24	NON SMALL CELL LUNG CANCER	*SRC1N1*	INVASION	(66)
25	HEAD AND NECK SQUAMOUS CELL CARCINOMA	*TGFβRII*	METASTASIS	(67)

## Oncogenic Effects of *MIR211*

Paradoxically, when *MIR211* expression was artificially induced in human melanoma cell lines, where its expression is generally reduced relative to those in melanocytes, which were xenografted into immunodeficient mice they resulted into aggressive tumor growth (68). Interestingly, this surprising observation is consistent with similar oncogenic effects associated with *MIR211* expression in a number of other cancer types, including oral carcinoma (65, 69), head and neck carcinoma (67), Burkitt lymphoma (70), breast cancer cell lines (71), and non-small-cell lung cancer (66).

There is no *a priori* reason why a regulatory molecule cannot be a tumor suppressor in one cancer type but oncogenic in another because the identity of differentially regulated downstream target genes in different cancer types might be the obvious mechanism. Indeed, separate sets of target genes of MIR211 have been shown to be responsible for its tumor-enhancing role (66, 69, 71). However, such an explanation does not easily explain how a regulatory molecule can be both a tumor suppressor and an oncogene in the same cancer type, such as seen with *MIR211* in melanoma. We propose below several mechanisms that should help resolve this important paradox.

## Cellular And/Or Genetic Contexts Determine the Effects of MIR211 Expression

We and others have reported that *MIR211* expression is reduced in amelanotic melanoma cell lines (12) and in clinical melanoma samples (44, 72), driving efforts to use *MIR211* as a clinical diagnostic test to discriminate melanomas from benign nevi (52). We also reported that the artificial over-expression of *MIR211* in amelanotic melanoma cells (A375) increases mitochondrial respiration by inhibiting *Pyruvate Dehydrogenase Kinase 4* (*PDK4*) mRNA levels, which was associated with reduced cell survival and lessened invasive properties under cell culture conditions (13). Consistently, miRNA profiling studies revealed that *MIR211* expression is down-regulated not only in melanoma (12, 54), but also in other cancers such as glioma (73) and glioblastoma (38), prostate cancer (74), hepatocellular carcinoma (48), epithelial ovarian cancer (45), cervical cancer (16, 56), and breast cancer (75). These foregoing results together point towards a general tumor-suppressor role of *MIR211*. By contrast, *MIR211* is also known exhibit high expression levels in certain melanoma subtypes and in other cancers: *MIR211* expression is high in a majority (6/8) of melanoma lines in the NCI-60 cancer cell panel (76), and in 9/29 clinical melanoma samples (12), suggesting that either MIR211 level by itself is irrelevant to the tumor-like status or malignancy or that in certain cancer cells MIR211 levels might have tumor-promoting activity.

The dual role of *MIRNA211* described above may hinge on the cellular context, similar to context-dependent regulation that was previously reported for other miRNA genes, in particular with *MIR7* (77, 78), *MIR125B* (79) and *MIR155* (80). This fascinating paradox—whether and how a particular miRNA could either inhibit or augment cancer growth and development depending on the particular cellular context—raises several questions concerning the importance of *MIR211* in melanocyte dedifferentiation (43), melanoma genesis (25), aging and senescence (81), and cardiac development (19).

**Figure 2:**
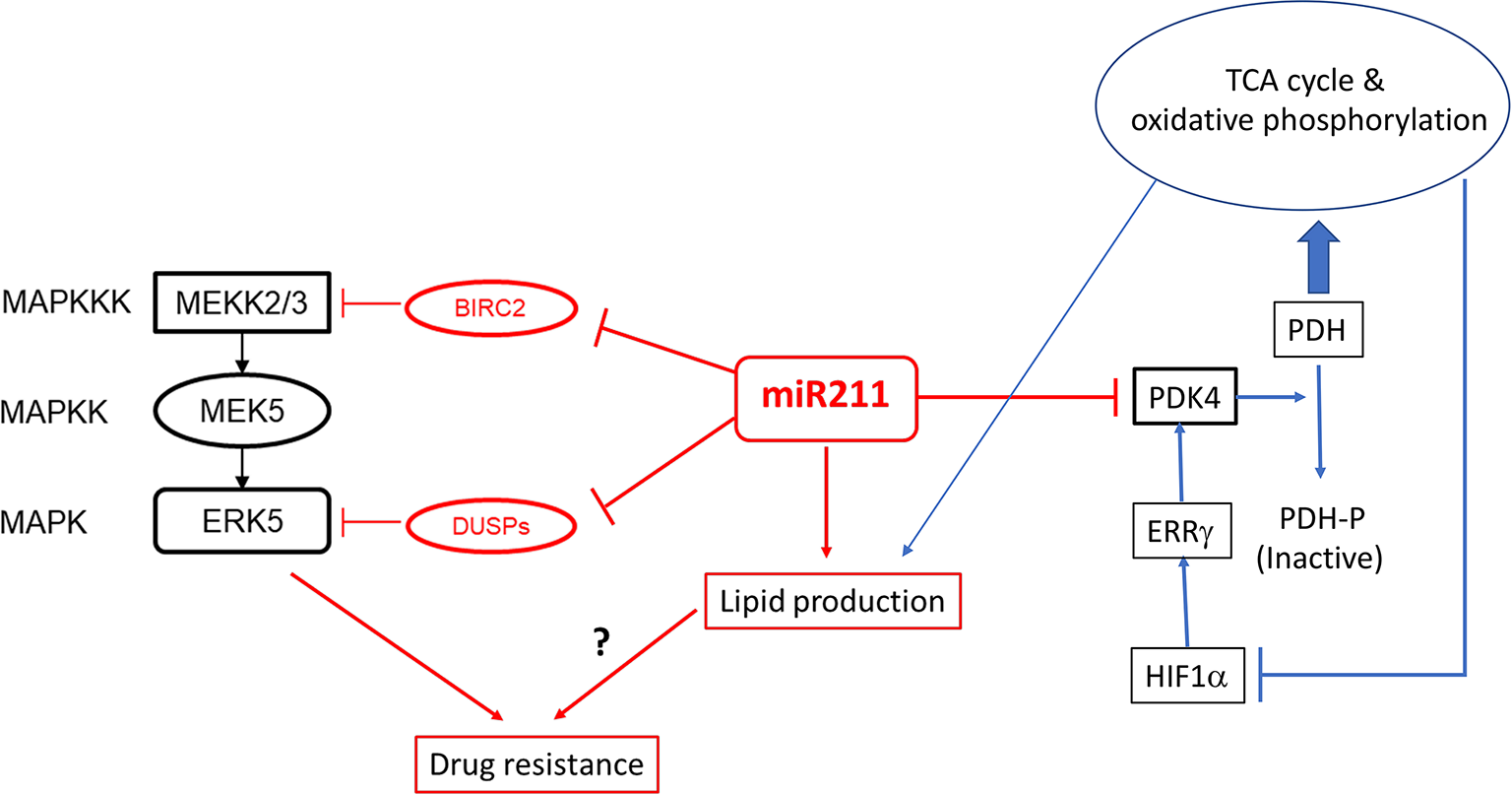
The regulatory cascade of MIR211 involving the BRAF/ERK5/DUSP and PDK4. MIR211 is a negative regulator of at least two negative regulators (BIRC2, DUSP6 and possibly other DUSP mRNAs) of the ERK5/MEK signaling pathway. The latter is involved in resistance of melanoma to vemurafenib and cobimetinib. PDK4 is a pyruvate dehydrogenase kinase, which inactivates pyruvate dehydrogenase (PDH) by phosphorylation, which therefore cannot catalyze the entry of pyruvate to tricarboxylic acid (TCA) cycle. Metabolites of the TCA cycle induces lipid biosynthesis and inhibits hypoxia inducing factor HIF1α, which is a transcriptional activator of ERRβ, which in turn is a transcriptional activator of *PDK4* gene. Evidence for these pathways are obtained from Lee *et al.* (2016 and 2020) (68, 82).

On the basis of the observations that MIR211 over-expression in the amelanotic melanoma cell line A375 decreased Warburg effect and simultaneously decreased cell proliferation and invasiveness (13), it was anticipated that these cells when transplanted as murine xenografts would also reduce tumor size relative to those by the control (normal MIR211 levels) A375 cells. Paradoxically, exactly the opposite results were observed (68), in which extraordinarily aggressive tumors were formed by the xenografted cells over-expressing MIR211. The *PDK4* gene, the target gene of MIR211 which was previously thought to be an effector of the metabolic switch controlled by MIR211, was indeed downregulated in these xenografts, and therefore the response of this target gene was unable to explain the paradoxical behavior of MIR211 on tumorigenesis. Deletion of *MIR211* gene in these xenografted cells produced much reduced tumors, supporting the conclusion that this MIR211 was directly responsible for the hypertrophic tumor phenotype. RNAseq analysis of mRNA molecules co-immunoprecipitated with the Argonaut-2 protein suggested that these mRNA molecules are associated with the RISC complex in cells with or without MIR211 (68). These studies further revealed several additional target mRNAs, including DUSP6 and BIRC2, which were then confirmed to be causally related to tumor hypertrophy *via* a modulation of the ERK5/MEK kinase signaling pathway (68) (**Figure 2**). These cells simultaneously became more resistant to the BRAF inhibitor vemurafenib and the MEK inhibitor cobimetinib, suggesting that the modulation of the DUSP6/ERK5 signaling pathway by MIR211 is related to the development of BRAF/MEK inhibitor resistant melanoma (68).

## Broadly Pleiotropic Tumor Suppressor and Oncogenic Behavior of MIR211 can be Explained By the Diversity of its Target mRNAs

As described above, MIR211 acts as a tumor suppressor in a variety of cancer types, it can also act as an oncogenic regulator in others. Therefore an understanding of the paradoxical behavior of MIR211 is of paramount importance for understanding cancer biology.

Though the functional role of MIR211 has not been studied extensively in primary human melanocytes, its tumor suppressor role in pigmented melanoma has reported widely and suggested to be due to a multitude of target genes [see citations above, and also (44, 83)]. Endoplasmic reticulum stress initiates the activation of three important signaling molecules like Ire-1α/β, ATF 6, and PERK, whose function is to enable adaptation to the ER stress. PERK (Eukaryotic translation initiation factor 2-alpha kinase 3, also known as protein kinase R (PKR)-like endoplasmic reticulum kinase), is an enzyme that in humans is encoded by the EIF2AK3 gene. PERK has been reported to induce the expression of MIR211, which in turn reduces the stress-dependent expression of GAD153/CHOP, a pro-apoptotic transcription factor at early stages of ER stress (84). If the ER stress is sustained, *MIR211* expression is turned off and increases the expression of CHOP and thereby apoptosis (84). A reduction in the expression of MIR211 in multiple myeloma is associated with increased expression of pro-apoptotic factor CHOP which in turn triggers ER stress mediated apoptosis (55). In epithelial ovarian cancer (EOC) cells, *MIR211* expression was down-regulated and increased the expression of cyclin D1 and CDK1 and thereby apoptosis (45). Levy et al. reported that MIR211 can directly inhibit the *TGFBR2* and *NFAT5* and can indirectly inhibit *IGF2R*, which leads to the reduced invasive potential of malignant melanomas (14).

EMT is a highly dynamic process that can convert nonmotile epithelial cells to motile mesenchymal cells (85), which involves molecular reprogramming and phenotypic changes that lead to tumor metastasis. EMT in cancer is associated with a decrease in the expression of epithelial cell markers such as γ-catenin and E cadherin, and with an increase in the expression of mesenchymal markers such as vimentin and N-cadherin (86). Mucin 4 (MUC4) is a glycoprotein that covers the epithelial cells and belongs to a large MUCIN family of glycoproteins. Mucin could be used as a lineage marker in benign cervical tissue, whose expression might be higher in cervical cancer (87). MIR211 could directly target MUC4 mRNA and thereby can inhibit the invasion and EMT in cervical cancer cells (57). In melanoma cells, MIR211 can modulate EMT by targeting RAB22A, a member of the RAB family of small GTPase, which regulates tumor invasion and metastasis in various cancer types (32).

Secreted protein acidic and rich in cysteine (SPARC) is a matricellular family of proteins, which modulates cell-matrix interactions and tissue remodeling and, thereby, EMT. In cervical cancer, MIR211 directly targets SPARC and thus acts as a tumor suppressor gene (16). MIR211 regulates Matrix metalloproteinase-9 (MMP-9) and thereby reduces EMT in gastric cancer (58). Sex-determining region Y-related high-mobility group box 4 (SOX4) is another crucial transcription factor that contributes to tumor cell survival, metastasis, and possibly to the maintenance of cancer stem cell properties. In gastric cancer cells, overexpression of MIR211 directly inhibits SOX4 expression and thereby down regulates tumor metastasis (88), suggesting the involvement of cancer stem cells. The Snail family transcriptional repressor 1 (SNAI1) has a significant role in regulating genes required for cell-cell interaction and EMT. By targeting SNAI1 mRNA, MIR211 directly regulates the metastatic behavior of tumor cells in renal cancer cells (49). PHF19 is a member of polycomb repressive complex 2 Complex, which mediates transcriptional repression of developmental regulatory genes and modulates embryonic stem cell differentiation. A recent report suggests that MIR211 can target PHF19 and thereby inhibit cell proliferation, migration and apoptosis in ovarian cancer (89). At the same time, MALAT1, a long non-coding RNA antagonizes the effect of MIR211 in ovarian cancer cells (46). The Secreted protein acidic and rich in cysteine (SPARC) is an oncogene, which is highly expressed in various tumors such as glioma, melanoma, prostate, and gastric carcinoma. SPARC belongs to the matricellular family and has a significant role in tissue repair and remodeling (90). MIR211 is a direct regulator of SPARC expression, and thereby down-regulates other metastasis-associated genes (62). ZEB1, a member of the zinc finger family protein, is reported to be upregulated in ovarian, breast, prostate, and endometrial cancer (91–94). MIR211 directly targets ZEB1 mRNA and reduces the proliferation and metastasis of cervical cancer cells (56).

Apart from regulating the apoptosis, Endoplasmic reticulum stress and EMT, MIR211 also regulates various other processes important for cancer, including angiogenesis, through influencing multiple signaling pathways. In hepatocellular carcinoma MIR211 down-regulates SATB2 (Human special AT-rich sequence-binding protein-2) and reduces its invasion and proliferation (48). Another important study demonstrated that in tongue squamous cell carcinoma the long non-coding RNA KCNQ1OT1 sponges MIR211 and thereby activates Ezrin/Fak/Src signaling pathways (95). Mazar *et al.* reported that MIR211 directly targets pyruvate dehydrogenase kinase 4 (*PDK4*) mRNA, and the ectopic expression of *MIR211* reduced hypoxia-inducible factor 1α (HIF-1α) protein levels and decreased cell growth during hypoxia (13). Ribonucleotide reductase M2 (RRM2) is associated with tumor progression and metastasis; and in colorectal cancer k–ras mutation reduces the expression of MIR211 which thereby enhances the expression of RRM2 (96). In melanotic melanoma cells, MIR211 is induced by BRAFi/MEKi and favors their pro-pigmentation activity by targeting EDEM1, hence promoting TYR expression and melanin accumulation (53). In another interesting study with breast cancer cells it was suggested that MIR211 targeted CDC25B, CCNB1, and FOXM1 and thereby inhibited cell cycle and reduced genomic stability, proliferation, migration, and invasion in triple-negative breast cancer cells (63). It was recently shown that adipocytes secret IL6, which leads to downregulation of MIR211 in melanoma which further promotes melanoma invasion (51).

In contrast to the tumor suppressor activities of MIR211 mentioned above, increased expression of MIR211 is reportedly oncogenic [see above, and (67, 97)]. Some of its oncogenic properties can also be traced to its target genes. For example, MIR211 appears to up-regulate cell cycle progression by targeting the tumor suppressor gene *CHD5* (64), thus modulating the p53 pathway, and *via* several protein kinases (98). MIR211 acts as an oncogene in Acute Myeloid Leukemia (97) by down-regulating the expression of *BIN1* and by activating the EGFR/MAPK pathway. Increased expression of MIR211 in head and neck squamous cell carcinoma (HNSCC) inhibited TGFβRII and thus decreased the SMAD3 phosphorylation and increased c-myc expression (67). MIR211 contributes to melanoma progression not only by targeting genes of melanoma cells but also by modulating the tumor niche in melanoma microenvironment *via* communication through melanosomes (15, 51). Melanosomal-MIR211 released from melanoma cells are transferred into the surrounding primary skin fibroblasts and which then induce their reprogramming into cancer-associated fibroblasts (CAFs) by targeting the IGF2R mRNA and through regulating MAPK signaling (15). The target genes of MIR211 which are considered to be important in various cancer types are summarized in **Table 1**.

## Paralogs of the MIR211 Family

A second *miRNA* gene that belongs to the same family as *MIR211* is *MIR204*, which is located within the *TRPM3* gene at 9q21.12, and is believed to be a paralog of *MIR211* (71). Both of these two mature MIRNAs share a similar seed sequence, but they differ in two nucleotides within their 3’ regions; these two MIRNAs share multiple common mRNA targets (99). Among these shared targets are the mRNAs of the following tumor-suppressing genes: *XIAP Associated Factor 1* (*XAF I*) (71, 100, 101), whose loss of function mutation causes gastric carcinoma, *HRAS-Like-Suppressor4* (or *HRASLS4*) (71, 101, 102), also associated with mutations in gastric cancer, *Homeodomain Interacting Protein Kinase 2* (*HIPK2*) (71, 103), mutations in which gene are associated with keratocanthoma (a rare skin cancer), breast cancer, and *Thioredoxin Interacting Protein* (*TXNIP*) (71, 104), associated with exocervical carcinoma. In the opposite direction, TargetScan (www.targetscan.org) analysis of MIR211-5p and MIR204-5p also yields the following common oncogene or oncogene-like genes as high-confidence targets: *Ras-Related Protein Rab-22A* (*RAB22A*), *Paired Like Homeobox 2B* (*PHOX2B*) whose high expression is associated with neuroblastoma (105), and *Adaptor Related Protein Complex 1 Subunit Sigma 2* (*AP1S2*) whose mRNA expression is enhanced in melanoma and is associated with significantly poorer survival of melanoma patients (although confers a better survival probability to ovarian and breast cancer patients). In summary, MIR211 and its paralog MIR204 together regulate a number of genes whose expression are related to both tumor suppressor and oncogenic activities in a number of different cancer types including melanoma. As a result, the complexity of phenotypes associated with *MIR211* could also be due to the combined as well as single negative effects on a number of different oncogenic and tumor suppressor mRNA levels, which at least in theory may produce rather complex net dynamics depending on the expression signatures of a large number of genes and the relative levels of the two microRNAs.

## Models Explaining the Paradoxical Effects of MIR211

The paradoxical behavior of MIR211 brings to mind the early controversy of p53, acting, as initially proposed, to promote tumor growth (106) but later understood to be a tumor suppressor (107). While as yet there is no direct relationship between p53 and MIR211, by reminding ourselves of the importance of paradoxes in science (108), especially concerning the p53 controversy, we suggest that MIR211 biology is important for understanding cancer biology.

**Figure 3:**
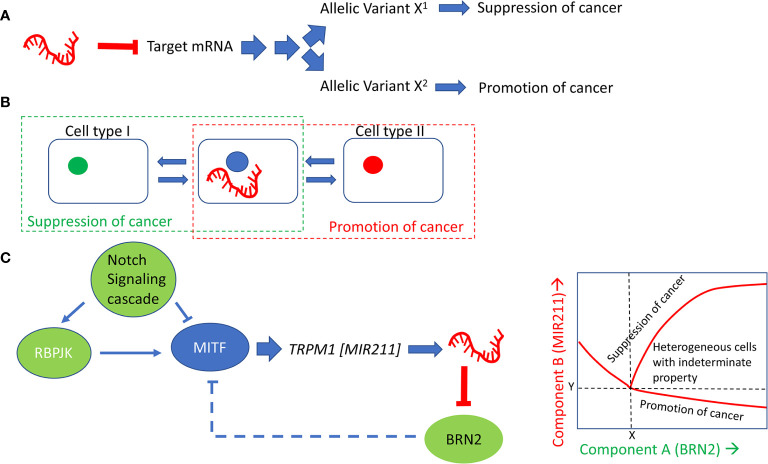
Models that explain the paradoxical effects of MIR211 on cancer development. **(A)** posits that allelic variants in MIR211 target genes explain the behavior of cells with respect to tumorigenesis. **(B)** assumes that the cellular type and the microenvironment, including cell-cell communication, determines the context for whether MIR211 will be tumorigenic or tumor suppressive. MIR211 secreted within exosomes, and/or signaling molecules or metabolites secreted by cells with high or low expression of MIR211 may influence adjacent tumor cells in concentration dependent manner, thus helping to establish distinct morphogenetic fields within the tumors and contribute to tumor heterogeneity. **(C)** explains the mutually opposite effects of MIR211 on the basis of cell to cell heterogeneity and dynamics of gene expression related to MIR211. In this last model, feed-forward and negative feedback with time delays set up opportunities for complex dynamical patterns of expression involving the two genes, *MIR211* and *BRN2*. The inset shows a schematic of such a dynamical pattern, in which stochastic fluctuation in the initial levels of MIR211 or BRN2 could lead to the transition at the parameter values (X,Y) of a cell to a tumor progression (high BRN2/low MIR211) or to a tumor suppressive (high BRN2/high MIR211) state and thus lock in the characteristic state. Intermediate states (high BRN2/intermediate MIR211) could represent cells that could be driven to either direction through other (environmental) factors, which has been shown to occur with melanoma cells with respect to their vemurafenib resistance phenotype (109).

There might be at least three different mechanisms that can explain the paradoxical behavior of a miRNA on a biological process: (a) differences in the allelic variants at miRNA targets; (b) differences in the cellular micro-environment, (c) stochastic fluctuations in the expression levels of the miRNA which set into motion different trajectories of regulatory influences in different cells. **Figure 3** summarizes the three models. It is unclear at this time which of these models is true of *MIR211*. These models are offered as the rationale to guide future experiments. The models are general, in the sense that these could formally apply to all cases where the same miRNA has multiple, mutually opposite, biological effects depending on the cellular contexts.

The allelic variant model (**Figure 3A**) proposes that nucleotide differences at the target site, especially within the seed sequences of the miRNA, are responsible for the cell-line to cell-line variability of its biological effects. Such variability might be inherited in the germline, or maybe due to somatic mutations. This model is consistent with several previous observations on miRNA target site sequence variations (110–112).

The micro-environment influence model (**Figure 3B**) posits that the expression of MIR211 triggers cell-nonautonomous events. Some cells in the tumor micro-environment sense the downstream effectors of MIR211, through either direct exosomal contribution of MIR211 to the neighboring cells or indirectly *via* cell-to-cell signaling and/or metabolites secreted by the primary cells. This proposal assumes the presence of morphogenetically heterogeneous cells within the tumor micro-environment, which takes into account the following previous observations: (i) Melanocytes are derived from non-pigmented melanocyte precursor stem cells, and non-pigmented melanomas are thought to be mainly derived from these stem cells through oncogenic transformation (113); (ii) pigmented melanoma cells are derived from dedifferentiation and transformation of melanocytes); (iii) *MIR211* expression is low in undifferentiated precursor cells, is high in melanocytes, low in non-pigmented melanoma, but high in pigmented melanoma cells (12); (iv) MIR211 targets PDK4, which causes high NADP^+^/NADPH ratio (13); (v) High NADP^+^/NADPH ratio causes high ROS levels (114). Diffusion of these molecules is in principle sufficient to set up distinct morphogenetic fields within the tumors (115).

The stochastic single-cell expression model (**Figure 3C**) posits that *MIR211* expression fluctuates stochastically from cell to cell (116), which leads to cell-to-cell fluctuations in the relative levels of *MITF* (component X) and *BRN2* (component Y). A well-known consequence of such fluctuations of two regulatory components in a mutually negative feedback regulatory loop is the establishment of multiple distinct equilibrium regions of expression levels of the two components in which they are both low, both high or one high/one low. Tumor gene expression systems have been shown to operate under such multiple equilibria, producing heterogenous cell populations within the tumors, leading to vastly different biological consequences (117). Importantly, the three models proposed here are not mutually exclusive; elements of each can coexist with those of the other two in defining the full range of biological complexity associated with this regulatory molecule.

## Summary and Recommendations

MIR211 shows both tumor suppressive and oncogenic activities in the same or different cancer types. Since MIR211 has also been shown to be associated with various human diseases apart from cancers, it is regarded as one of the most promising miRNA molecules for therapeutic applications. To our knowledge no clinical trial is currently on-going with this miRNA. Although numerous studies have illuminated the molecular mechanisms associated with the tumor-suppressive and oncogenic characteristics of MIR211, there are many important questions that need to be answered in the future, and we have provided three different frameworks for approaching these mechanisms. We suggest that further work on this interesting regulatory molecule should focus on (a) deciphering the source of mutually opposite behavior of MIR211 in cancer, (b) understanding the mechanisms of cell-to-cell expression variances of *MIR211*, and (c) how such variations could be utilized for diagnostic, prognostic or therapeutic applications.

## Author Contributions

AR and RP incepted the idea on the paradoxical behavior of the MIR-211 in melanoma and other cancers. All authors (AR, RP, and HK) contributed to the writing. All authors contributed to the article and approved the submitted version.

## Funding

This study was supported by P30 CA006973 (JHU SKCCC) and Florida Department of Health, Bankhead-Coley Cancer Research Program (5BC08) to RP, and CDMRP/DTRA grant W81XWH-16-1-0170 to AR.

## Conflict of Interest

The authors declare that the research was conducted in the absence of any commercial or financial relationships that could be construed as a potential conflict of interest.

## Footnotes

1 We use the convention for human miRNAs as proposed in (1), where a human miRNA is MIRNA followed by a number, and its corresponding gene is italicized.
